# Acute physiological and psychological responses during an incremental treadmill test wearing a new upper-body sports garment with elastomeric technology

**DOI:** 10.3389/fphys.2024.1372020

**Published:** 2024-04-22

**Authors:** Danica Janicijevic, Angel Saez-Berlanga, Carlos Babiloni-Lopez, Fernando Martin-Rivera, Pablo Jiménez-Martínez, Alejandro Silvestre-Herrero, Javier Martínez-Puente, Pablo Ferradás-Nogueira, Alvaro Juesas, Javier Gene-Morales, Iván Chulvi-Medrano, Juan C. Colado

**Affiliations:** ^1^ Faculty of Sports Science, Ningbo University, Ningbo, China; ^2^ Department of Radiology, Ningbo No. 2 Hospital, Ningbo, China; ^3^ Department of Sports Sciences and Physical Conditioning, Faculty of Education, Universidad Catolica de la Santísima Concepción, Concepción, Chile; ^4^ Research Group in Prevention and Health in Exercise and Sport (PHES), Department of Physical Education and Sports, University of Valencia, Valencia, Spain; ^5^ ICEN Institute, Madrid, Spain

**Keywords:** treadmill test, variable resistance training, muscle activation, metabolic and cardiovascular responses, physical performance

## Abstract

**Background:** The use of elastomeric technology in sports garments is increasing in popularity; however, its specific impact on physiological and psychological variables is not fully understood. Thus, we aimed to analyze the physiological (muscle activation of the pectoralis major, triceps brachii, anterior deltoid, and rectus abdominis, capillary blood lactate, systolic and diastolic blood pressure, and heart rate) and psychological (global and respiratory rating of perceived exertion [RPE]) responses during an incremental treadmill test wearing a new sports garment for the upper body that incorporates elastomeric technology or a placebo garment.

**Methods:** Eighteen physically active young adults participated in two randomized sessions, one wearing the elastomeric garment and the other wearing a placebo. Participants performed in both sessions the same treadmill incremental test (i.e., starting at 8 km/h, an increase of 2 km/h each stage, stage duration of 3 min, and inclination of 1%; the test ended after completing the 18 km/h Stage or participant volitional exhaustion). The dependent variables were assessed before, during, and/or after the test. Nonparametric tests evaluated differences.

**Results:** The elastomeric garment led to a greater muscle activation (*p* < 0.05) in the pectoralis major at 16 km/h (+33.35%, *p* = 0.01, *d* = 0.47) and 18 km/h (+32.09%, *p* = 0.02, *d* = 0.55) and in the triceps brachii at 10 km/h (+20.28%, *p* = 0.01, *d* = 0.41) and 12 km/h (+34.95%, *p* = 0.04, *d* = 0.28). Additionally, lower lactate was observed at the end of the test (−7.81%, *p* = 0.01, *d* = 0.68) and after 5 min of recovery (−13.71%, *p* < 0.001, *d* = 1.00) with the elastomeric garment. Nonsignificant differences between the garments were encountered in the time to exhaustion, cardiovascular responses, or ratings of perceived exertion.

**Conclusion:** These findings suggest that elastomeric garments enhance physiological responses (muscle activation and blood lactate) during an incremental treadmill test without impairing physical performance or effort perception.

## 1 Introduction

The literature shows that endurance exercise confers health and performance benefits such as improving or maintaining cardiovascular fitness and body composition (Gibala and Jones, 2013; Milanović et al., 2015; Wewege et al., 2017). Achieving an optimal training stimulus is key to obtaining and maximizing these benefits of exercise (Weston et al., 2014; Milanović et al., 2015). Recently, new sports tools (e.g., compression garments, gravity vests, and weight vests) have appeared to enhance training stimuli, and scientists have examined their potential positive effects on physically active individuals and professional athletes (Cipriani et al., 2014; Marriner et al., 2017; Feser et al., 2021). For instance, employing a compressive garment during exercise likely improves performance due to possible physiological effects (Marqués-Jiménez et al., 2018; Williams et al., 2021). Additionally, elastic devices have emerged as plausible materials to increase physical capacities because they are transportable, affordable, and have proven positive results (Gene-Morales et al., 2020; Babiloni-Lopez et al., 2022; Gene-Morales et al., 2022; Hammami et al., 2022; Saez-Berlanga et al., 2022). These devices have been commonly employed in strength training, and depending on how they are applied, they can both assist and resist movements. Through the use of elastics to resist movement, the participants achieved more muscle activation (Argus et al., 2011; Upton, 2011). Furthermore, the use of elastics as assistance also makes it possible to mobilize a load at a higher velocity (Tran et al., 2012). Taking this into consideration, the question arises as to whether new state-of-the-art tools can optimize the training stimulus. To our knowledge, there are still no scientific studies that examined the effects of performing an incremental treadmill test with a sports garment that incorporates elastomeric technology on parameters of the internal and external load.

In this regard, it is important to know how the use of an elastomeric garment can influence different aspects of physical performance and physiological and psychological responses. First, time to exhaustion is a key parameter of running performance in an incremental treadmill test (Scheer et al., 2018). Second, it is necessary to monitor muscle activity including surface electromyography (EMG), which records the electrical activity of the peripheral nervous system during muscle contractions (Hermens et al., 2000). This measure depends on the neuronal signals sent from motor neurons to muscles (Farina and Enoka, 2023). In this regard, afferent and efferent signals from the nervous system during skeletal muscle contraction are key to understanding exercise adaptations (Alix-Fages et al., 2022). Likewise, the metabolic and cardiovascular components of performance can alternatively be examined using metabolites such as blood lactate concentration and hemodynamic responses (e.g., blood pressure and heart rate) (Beneke et al., 2011; Gripp et al., 2021). Lactate concentration analysis is a valid option for quantifying training intensity, with blood lactate levels increasing in response to high-intensity training (Cerezuela-Espejo et al., 2018). Finally, it is crucial to determine whether the use of the elastomeric garment influences psychological variables (e.g., rating of perceived exertion [RPE]). Prior studies have informed that the RPE is correlated with various exercise-related factors such as heart rate and blood lactate (Scherr et al., 2013). In light of this, numerous subjective scales have been validated to assess and confirm the intensity of different types of exercise with various training devices, including elastic bands (Colado et al., 2012; Colado et al., 2014; Colado et al., 2018; Colado et al., 2020; Colado et al., 2023).

Therefore, this study aimed to evaluate: i) running performance (time to exhaustion); ii) physiological (muscle activation, capillary blood lactate concentration, blood pressure, and heart rate); and iii) psychological (global and respiratory rating of perceived exertion) responses during an incremental treadmill test performed wearing or not a new sports garment for the upper body that incorporates elastomeric technology. Considering that depending on whether the elastomers are shortened or stretched, the garment can offer extra resistance or assistance (Saez-Berlanga et al., 2024), we hypothesized that the use of this garment would optimize muscle activation levels in all of the muscles tested, without modifications in capillary blood lactate concentration, hemodynamic responses, perceived exertion rating, and time to exhaustion.

## 2 Materials and methods

### 2.1 Participants

The sample size was determined using G*Power 3.1 software (Faul et al., 2007) based on previous studies (Gene-Morales et al., 2023). An *a priori* analysis was conducted to minimize the probability of type II error and calculate the minimum number of participants needed to reject the null hypothesis at the *p* < 0.05 level of confidence (Beck, 2013). The calculation showed that 18 volunteers were required to achieve a power of 0.80, a significance level of 0.05, and an effect size f(V) of 0.83.

The participants involved in the project were thoroughly briefed on the possible risks and discomfort associated with it, and they willingly signed a written consent form before the commencement of the study. The study protocol was approved by the Human Research Ethics Committee of the University of Valencia (H20190325095509 and H1460994903890) and was conducted in compliance with the principles outlined in the Declaration of Helsinki.

The study consisted of young adults aged 18–30 years with experience in training (at least three sessions of physical activity per week). These participants were required to be free of any history of osteoarticular or cardiovascular disease and of any cognitive, clinical, or neuromotor contraindications that would prevent them from performing the physical test. A total of 18 physically active men were selected through convenience sampling and willingly participated in the study. The participants were instructed to refrain from consuming any food, stimulants (e.g., energy drinks or coffee), or other ergogenic substances 3 hours before the sessions and to avoid engaging in intense physical activity or exercise for the lower limbs 24 h before the study. They were also encouraged to maintain good quality sleep the night before data collection.

### 2.2 Procedures

**FIGURE 1:**
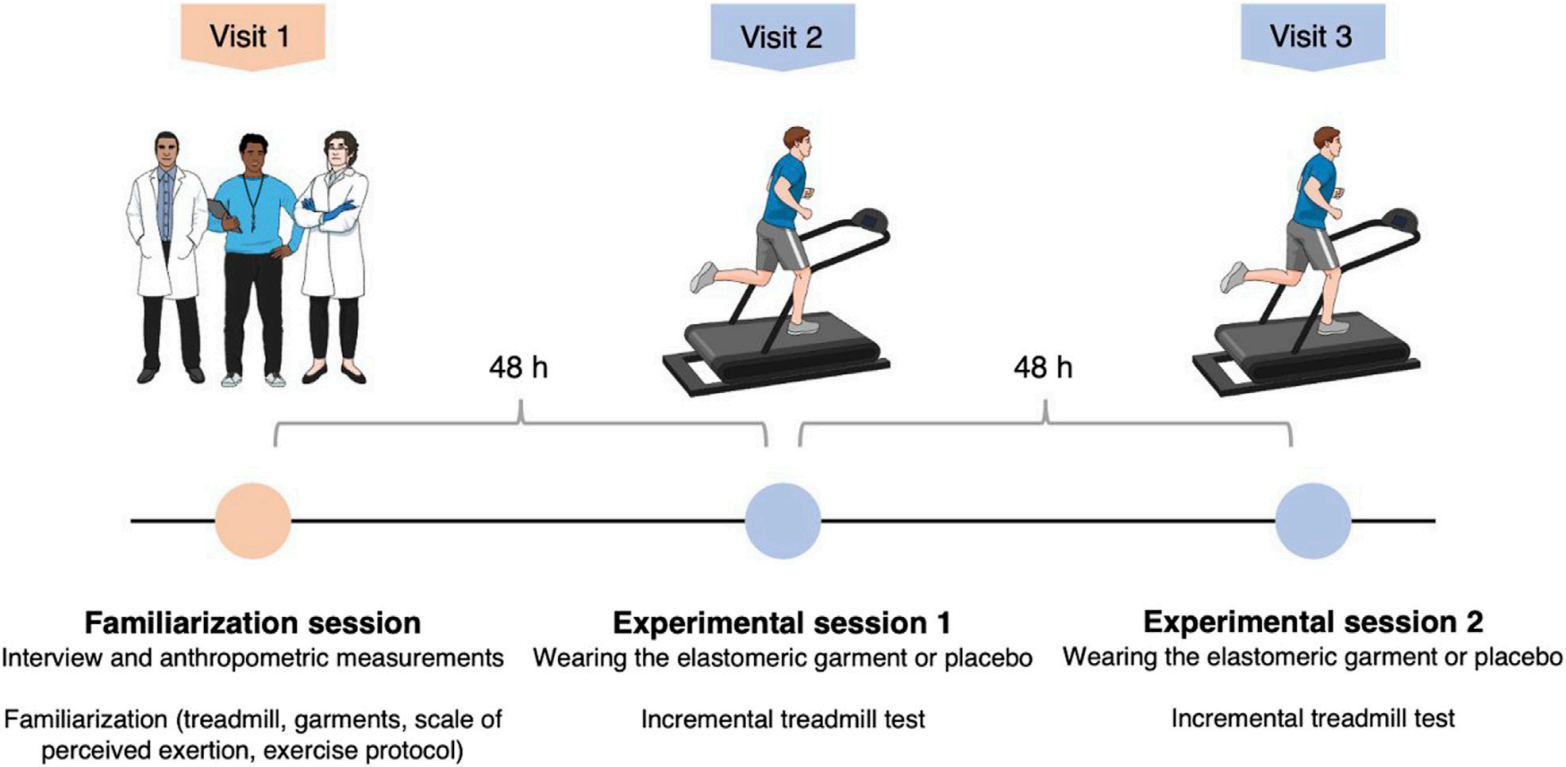
Schematic diagram of the experimental protocol.

A within-participants randomized crossover study design was used to examine the effects of treadmill exercise testing using a novel upper-body sports garment that incorporates elastomeric technology. Each participant was involved in three sessions 48 h apart, one of which was a familiarization and initial assessment, two of which were experimental sessions. The overall study design is illustrated in Figure 1. All sessions were conducted between 9:00 a.m. and 1:00 p.m. to avoid diurnal fluctuations in the performance or the other dependent variables. Each participant completed both experimental sessions within the same hour. The study was conducted over 8 weeks at the Physical Activity and Health Laboratory of the University of Valencia (Spain). All measurements were performed by the same researchers at the same sports laboratory. The minimum researchers-to-participant ratio was 4:1.

#### 2.2.1 Familiarization

The familiarization session was used to (i) characterize the participants through anthropometric measurements and interviews; (ii) use the two sports garments (i.e., placebo and elastomeric garment); (iii) show and explain the operation of the treadmill as well as the incremental testing protocol; and (iv) instruct participants on how to report the overall body and respiratory rating of perceived exertion (RPE) at the end of each stage of the treadmill exercise test.

After a pre-exercise interview, anthropometric measurements, body weight, and fat percentage were obtained using a bioimpedance analyzer (Tanita BF-350, Tanita Corp., Tokyo, Japan). The height was determined to the nearest 0.5 cm during maximum inhalation using a wall stadiometer (Seca T214, Seca Ltd., Hamburg, Germany).

The participants were familiarized with the instruments used throughout the study, including a treadmill (ExciteLiveRun; Technogym, Cesena, Italy). To assess global and respiratory RPE, a modified Borg Scale 0–10 (Borg, 1982) was used, which was visible to the participants during the exercise. Finally, the participants were shown the two garments to use during the study: a sports garment with elastomeric technology (Pro-advance, Menatechpro System^®^, Madrid, Spain), and a placebo garment that appeared identical but did not include elastomers. Menatechpro System^®^ elastomeric technology is a patented, advanced sportswear that generates elastic resistance in most planes of motion through the elongation of elastomers included in the garment. Specifically, the garment features front and back elastomers around the chest, which connect to the shoulders and extend down to the hands through each arm with two lines of elastomers. Additionally, due to this specific disposition of the elastomers, this garment could also assist in certain parts of the range of motion. Therefore, the garment’s ability to offer resistance, and/or assistance for diverse movements is determined by the way its elastomers are arranged. Furthermore, the elastomeric garment due to its characteristics can provide compression to the upper body. The Pro-advance model, which was employed in the current research, offers a maximum resistance of 8 kg at its maximum elongation, making it suitable for users who have prior training experience and aim to enhance their exercise intensity. Twelve similar garments (i.e., six placebo garments and six garments with elastomeric technology) were utilized for the study for a better adaptation of the garment to the anthropometric characteristics of each participant.

Subsequently, the participants were instructed to perform the standardized warm-up, which consisted of dynamic stretching, bodyweight strength exercises (e.g., lunges and squats), and a treadmill run of 5 min (starting at 7 km/h and increased by 1 km/h each minute) (Scheer et al., 2018). Participants were provided with a detailed explanation of the modified Borg scale (0–10) (Borg, 1982), where 10 was considered “maximum effort” and 0 “no effort.” Afterward, we explained the development of the test following the protocol of the study by Scheer et al. Scheer et al. (2018): starting at 8 km/h, an increase of 2 km/h each stage, stage duration of 3 min, and inclination of 1%. The test ended after the participant completed the 18 km/h Stage or at volitional exhaustion. A standardized pause of 30 just at the end of the second stage was implemented for capillary blood lactate measurement. The participants were instructed to self-manage the running technique according to their characteristics and experience. In addition, the participants received constant verbal and visual feedback to maintain proper body position during the test.

#### 2.2.2 Experimental sessions

The study comprised two experimental sessions, one of which was conducted entirely with the elastomeric garment, while the other was conducted with a placebo garment. The order of the sessions was randomly assigned to each participant in the first session using a randomizer (https://random.org/lists). Both sessions consisted of performing the incremental treadmill exercise test as previously explained. Upon arrival at the laboratory in both experimental sessions, participants rested in a seated position for 10 min while listening to self-selected music to induce similar resting homeostatic conditions between the sessions.

In the first experimental session, participants performed the standardized warm-up, and after a 5-min rest, the participants executed the incremental treadmill exercise test. The neuromuscular activation of each muscle group was recorded during the first minute of each stage. The EMG measures started in the 10 km/h Stage. The capillary blood lactate concentration was evaluated at the end of the second stage to gauge intra-effort levels. At the end of each stage, the participants verbally mentioned the RPE as they ran. Immediately after the treadmill exercise test, blood lactate concentration and cardiovascular variables such as heart rate and systolic and diastolic blood pressure were measured. These measurements were conducted with the participant seated in an adjoining space separated from the exercise area by a partition to conceal the procedures. Intra-test analysis was performed with the participants standing with their feet placed on the borders of the treadmill. The same procedure was followed in the second experimental session.

### 2.3 Measurement equipment and data acquisition

#### 2.3.1 Time to exhaustion

We recorded the time to exhaustion with a stopwatch. The timing was stopped when the participant’s hands touched the treadmill frame, or a verbal request to stop the test was made.

#### 2.3.2 Physiological variables: neuromuscular activation

The EMG signal was obtained using two synchronized two-channel handheld devices (Realtime Technologies Ltd., Dublin, Ireland) with a 16-bit analog-to-digital (A/D) conversion. EMG data were monitored using the validated (Hermens et al., 2000) mDurance software for Android (mDurance Solutions S.L., Granada, Spain). To ensure consistency in the standardized electrode placement, we followed the Surface Electromyography for the Non-Invasive Assessment of Muscles criteria (SENIAM) (Hermens et al., 2000) and previous studies in this field (Calatayud et al., 2014; Calatayud et al., 2017). Each participant was required to shave and clean the designated area using a cotton swab moistened with alcohol. Surface electrodes were placed on the anterior deltoid, clavicular fibers of the pectoralis major, upper rectus abdominis, and long head of the triceps brachii, according to previous studies (Calatayud et al., 2014). Chlorinated silver pre-gelled bipolar surface electrodes (Kendall™ Medi-Trace, Coividien, Barcelona, Spain) were placed at an inter-electrode distance of 10 mm. The reference electrode was positioned over the nearest bone prominence (in our study, the acromion and the superior iliac spine). To ensure consistent placement and reliable data, a mark was made on the skin of the participants around each electrode, using a permanent marker. One device recorded EMG data from the anterior deltoid and clavicular bundles of the pectoralis major muscle, while the other collected data from the upper rectus abdominis and long head of the triceps brachii muscles. The sampling rate was set at 1,024 Hz.

EMG signals were recorded and stored on a hard disk for subsequent analysis. The mDurance software used a fourth-order “Butterworth” bandpass filter to automatically filter the raw signals between 20 and 450 Hz. A high-pass cut-off frequency of 20 Hz was applied to minimize any potential “artifacts” that may occur during movement and affect the total power recorded by the EMG. Before the tests, the participants performed dynamic gestures typical of running to ensure proper signal saturation. From the second stage of the test (10 km/h), during the first minute of each stage, the average EMG signals (measured by the root-mean-square [RMS] value in microvolts) were collected for analysis. In the case that any of the participants did not complete integrally the first minute of the last stage, the EMG signal was collected for the time spent running during that last stage.

#### 2.3.3 Physiological variables: capillary blood lactate

Blood lactate concentrations were measured from capillary blood obtained from the fingertips. Blood samples were collected (i) before the test, (ii) at the end of the second stage of the test (intra-test), (iii) immediately after the treadmill exercise test, and (iv) 5 min after the end of the test. The samples were analyzed using a portable lactate analyzer (Lactate Pro 2, Arkray Inc., Kyoto, Japan).

#### 2.3.4 Physiological variables: cardiovascular parameters

Heart rate and systolic and diastolic blood pressure were assessed before and after the test using a digital wrist blood pressure monitor (RS4-model; Omron Electronics Iberia SAU, Valencia, Spain).

#### 2.3.5 Psychological variables: rating of perceived exertion

Overall body and respiratory RPE were measured using a modified Borg Scale 0–10 (Borg, 1982). The scale was visible to participants throughout the sessions. To rate the effort participants had to answer at the end of each stage: “How hard does it feel now to run?” “How hard does it feel now to breathe?” “How intense is your overall sensation of breathing?” or “How intense is your sensation of unsatisfied inspiration?” (Aliverti et al., 2011; Lewthwaite and Jensen, 2021).

### 2.4 Statistical analyses

Statistical analyses were performed using the commercial software IBM SPSS (version 28.0; IBM Corp., Armonk, New York, United States). The results were reported as the mean ± standard deviation. A 95% confidence level (significance *p* ≤ 0.05) was considered statistically significant. The Shapiro–Wilk test was used to ascertain a normal distribution of the variables. All variables showed a non-normal distribution except time to exhaustion.

Therefore, differences between conditions (elastomeric garment or placebo) in time to exhaustion were assessed using a *t*-test. Additionally, Friedman’s test was used to assess the effects of time (measurements pre-, intra-, and post-test) in both conditions. The effect size for the Friedman test was assessed using Kendall’s coefficient of concordance (W) and interpreted according to Cohen’s guidelines (Cohen, 2009). Afterward, Wilcoxon’s comparisons were performed to assess differences between each time point and between conditions (elastomeric garment and placebo) at each time point. Effect sizes were calculated using Cohen’s *d* as previously described (Cohen, 2009). Cohen’s *d* was interpreted as a trivial effect (<0.20), small effect (0.20–0.50), moderate effect (0.50–0.80), and large effect (>0.80) (Cohen, 2009).

## 3 Results

### 3.1 Participants

Eighteen healthy and trained participants were included in the study. None of the participants were excluded from this study. Descriptive data of the participants in this study were: 24.3 ± 4.4 years; height: 179.1 ± 4.1 cm; body mass: 76.4 ± 8.5 kg; body fat: 13.7% ± 4.6%; weekly training frequency: 3.6 ± 1.1 days/week.

### 3.2 Time to exhaustion

The mean time to exhaustion in seconds for the incremental treadmill exercise test performed with the elastomeric garment and placebo garment was 1015.00 ± 95.56 and 1011.66 ± 93.06 s, respectively. The t-test revealed no significant differences between using the elastomeric garment or the placebo (*p* = 0.854).

### 3.3 Physiological variables: neuromuscular activation

Descriptive and inferential analyses of the neuromuscular activation outcomes included in the study are presented in Table 1.

**TABLE 1:** Neuromuscular activation outcomes to incremental treadmill test performed wearing the elastomeric garment or the placebo.

	Elastomeric garment					Placebo garment				
	*Stage 10* * * *km/h*	*Stage 12* * * *km/h*	*Stage 14* * * *km/h*	*Stage 16* * * *km/h*	*Stage 18* * * *km/h*	*Stage 10* * * *km/h*	*Stage 12* * * *km/h*	*Stage 14* * * *km/h*	*Stage 16* * * *km/h*	*Stage 18* * * *km/h*
*RMSpec (μV)*	17.53 (9.67)^a^ ^,^ ^b^ ^,^ ^c^ ^,^ ^d^	21.94 (10.97)^b^ ^,^ ^c^ ^,^ ^d^	24.10 (13.75)^d^	26.15* (14.50)^d^	40.75* (20.20)	14.81 (5.27)^a^ ^,^ ^b^ ^,^ ^c^ ^,^ ^d^	18.11 (5.41)^d^	19.43 (5.79)^d^	19.61 (6.37)^d^	30.85 (8.38)
*RMStri (μV)*	23.78* (13.23)^a^ ^,^ ^c^ ^,^ ^d^	33.94* (14.12) ^d^	25.67 (6.77)^d^	27.73 (7.84)^d^	40.29 (8.05)	19.77 (10.50)^a^ ^,^ ^b^ ^,^ ^c^ ^,^ ^d^	25.15 (13.51)^b^ ^,^ ^d^	26.83 (12.90)^d^	27.73 (11.95)^d^	39.85 (14.44)
*RMSdelt (μV)*	10.13 (2.81)^a^ ^,^ ^b^ ^,^ ^c^ ^,^ ^d^	14.29 (4.93) ^c^ ^,^ ^d^	15.56 (5.85)^c^ ^,^ ^d^	19.03 (7.93)^d^	34.97 (9.97)	12.59 (8.09)^a^ ^,^ ^b^ ^,^ ^c^ ^,^ ^d^	16.95 (9.39)^b^ ^,^ ^c^ ^,^ ^d^	18.79 (9.74)^d^	18.37 (10.07)^d^	35.19 (10.46)
*RMSabd (μV)*	39.36 (16.63)^a^ ^,^ ^b^ ^,^ ^c^ ^,^ ^d^	49.73 (17.18) ^d^	47.00 (16.42)^d^	48.06 (11.99)^d^	65.16 (14.41)	36.02 (14.91)^a^ ^,^ ^b^ ^,^ ^c^ ^,^ ^d^	38.97 (12.67)^c^ ^,^ ^d^	39.67 (10.97)^c^ ^,^ ^d^	41.98 (11.68)^d^	59.65 (11.74)

Results are presented as mean ± standard deviation.

^a^ Significant differences (*p* < 0.05) compared with Stage 12 km/h.

^b^ Significant differences compared with Stage 14 km/h.

^c^ Significant differences compared with Stage 16 km/h;

^d^ Significant differences compared with Stage 18 km/h; *Significant differences compared with the placebo garment; μV: microvolts; RMSpec: root-mean-square pectoralis major; RMStri: root-mean-square triceps brachii; RMSdelt: root-mean-square anterior deltoid; RMSabd: root-mean-square rectus abdominis.

The factor time showed a significant effect on all the neuromuscular activation parameters both wearing the elastomeric garment (RMSpec, χ2 = 34.47, *p* < 0.001, W = 0.73; RMSdelt, χ2 = 41.67, *p* < 0.001, W = 0.87; RMStri, χ2 = 25.27, *p* < 0.001, W = 0.53; RMSabd, χ2 = 26.20, *p* < 0.001, W = 0.55) and placebo garment (RMSpec, χ2 = 39.80, *p* < 0.001, W = 0.83; RMSdelt, χ2 = 35.40, *p* < 0.001, W = 0.74; RMStri, χ2 = 32.47, *p* < 0.001, W = 0.68; RMSabd, χ2 = 30.93, *p* < 0.001, W = 0.64).

Wearing the elastomeric garment to perform the treadmill exercise test entailed greater RMSpec during the last two stages of the test (16 km/h, *p* = 0.01, *d* = 0.47; 18 km/h, *p* = 0.02, *d* = 0.55) and RMStri during the first two stages of the test (10 km/h, *p* = 0.01, *d* = 0.41; 12 km/h, *p* = 0.04, *d* = 0.28), compared to the same test performed with the placebo garment. Non-significant differences between using the elastomeric garment or the placebo were found in RMSdelt (all *p* ≥ 0.19, *d* ≤ 0.33) and RMSabd (all *p* ≥ 0.09, *d* ≤ 0.43).

### 3.4 Physiological variables: capillary blood lactate

A significant effect of time was found in the capillary blood lactate concentration (elastomeric garment, χ2 = 52.87, *p* < 0.001, W = 0.98; placebo garment, χ2 = 48.79, *p* < 0.001, W = 0.90).

**FIGURE 2:**
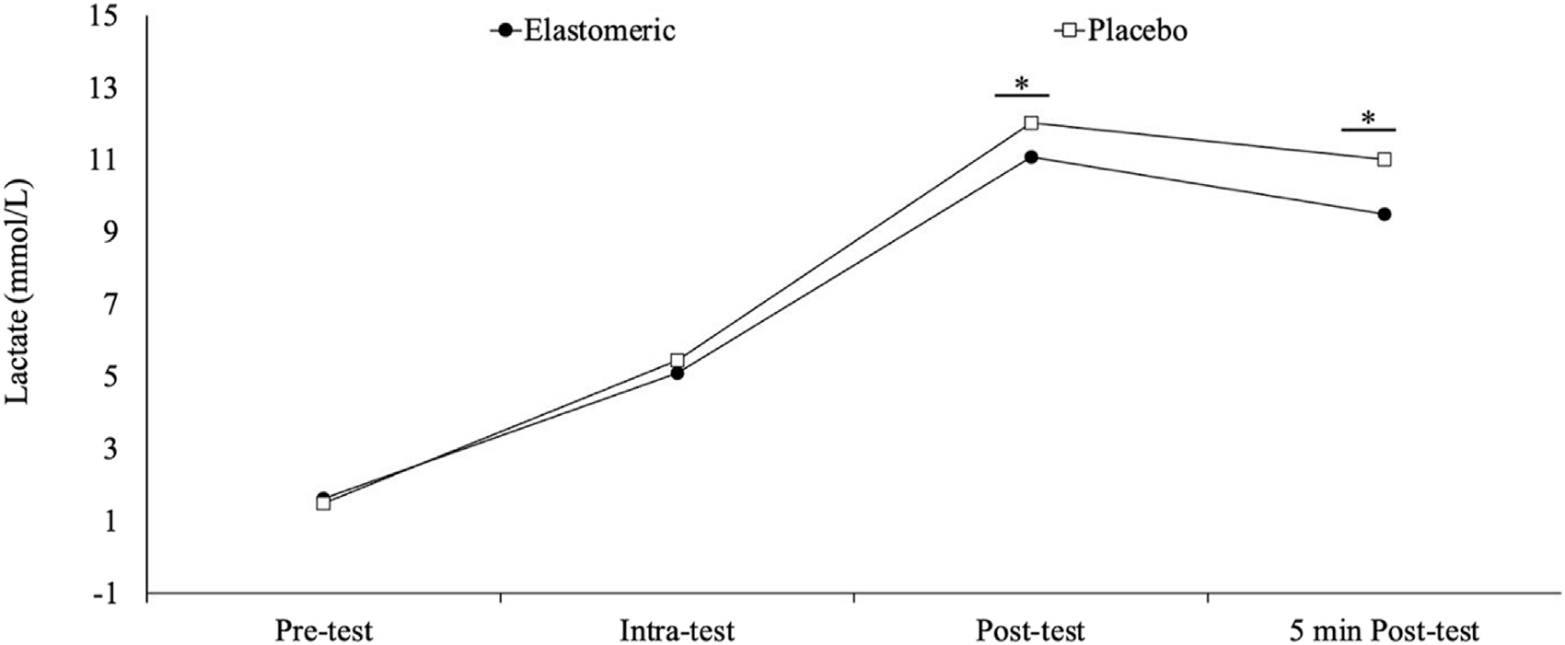
Variation of the capillary blood lactate through the incremental treadmill test performed wearing the elastomeric garment or the placebo. * Denotes significant difference from conditions (*p*<0.05).

As displayed in Figure 2; Table 2, wearing the elastomeric garment entailed significantly lower capillary blood lactate at the end of the test (*p* = 0.01, *d* = 0.68) and 5 min after the end of the test (*p* < 0.001, *d* = 1.00) compared to the placebo. Non-significant differences between garments were found during the test (*p* = 0.23, *d* = 0.36).

**TABLE 2:** Capillary blood lactate and cardiovascular outcomes to incremental treadmill test performed wearing the elastomeric garment or placebo.

	Elastomeric garment				Placebo garment			
	*Pre-test*	*Intra-test*	*Post-test*	*5* * *min *post-test*	*Pre-test*	*Intra-test*	*Post-test*	*5* * *min *post-test*
*Blood lactate* (*mmol/L*)	1.62 (0.43)^a^ ^,^ ^b^ ^,^ ^c^	5.09 (1.06)^b^ ^,^ ^c^	11.09* (2.34)^c^	9.50* (2.10)	1.48 (0.46)^a^ ^,^ ^b^ ^,^ ^c^	5.45 (1.29)^b^ ^,^ ^c^	12.03 (2.57)^c^	11.01 (2.40)
*Heart rate* (*bpm*)	66.83 (11.74)^b^		129.28 (21.21)		65.00 (8.36)^b^		130.44 (20.35)	
*Systolic blood pressure* (*mmHg*)	125.67 (12.42)^b^		144.94 (11.12)		125.39 (15.53)^b^		147.61 (23.27)	
*Diastolic blood pressure* (*mmHg*)	83.44 (9.01)^b^		100.33 (12.22)		85.06 (12.53)^b^		103.17 (21.25)	

Results are presented as mean ± standard deviation.

^a^ Significant differences (*p* < 0.05) compared with the intra-test.

^b^ Significant differences compared with the post-test.

^c^ Significant differences compared with the 5 min post-test; *Significant differences compared with the placebo garment; mmol/L: millimole per liter; bpm: beats per minute; mmHg: millimeters of mercury.

### 3.5 Physiological variables: heart rate

We observed a significant effect of time on the heart rate (elastomeric garment, χ2 = 18.00, *p* < 0.001, W = 1.00; placebo garment, χ2 = 18.00, *p* < 0.001, W = 1.00).

No differences between conditions (elastomeric garment, placebo) were found in the heart rate (*p* = 0.67, *d* = 0.10) (Table 2).

### 3.6 Physiological variables: systolic and diastolic blood pressure

The factor time showed a significant effect on the blood pressure (elastomeric garment: systolic blood pressure, χ2 = 14.22, *p* < 0.001, W = 0.79; diastolic blood pressure, χ2 = 10.89, *p* < 0.001, W = 0.61; placebo garment: systolic blood pressure, χ2 = 4.76, *p* = 0.02, W = 0.27; diastolic blood pressure, χ2 = 8.00, *p* = 0.005, W = 0.44).

We encountered non-significant differences in the diastolic (*p* = 0.58, *d* = 0.17) and systolic blood pressure (*p* = 0.48, *d* = 0.15) between using the elastomeric garment or the placebo (Table 2).

### 3.7 Psychological variables: rating of perceived exertion

A significant effect of time was found in the global RPE (elastomeric garment, χ2 = 64.51, *p* < 0.001, W = 0.99; placebo garment, χ2 = 64.67, *p* < 0.001, W = 0.99) and respiratory RPE (elastomeric garment, χ2 = 64.36, *p* < 0.001, W = 0.99; placebo garment, χ2 = 64.60, *p* < 0.001, W = 0.99).

Non-significant differences were encountered in the global RPE (all *p* ≥ 0.06, *d* ≤ 0.49) and respiratory RPE (all *p* ≥ 0.09, *d* ≤ 0.41) between using the elastomeric garment or the placebo (Table 3).

**TABLE 3:** Psychological responses to incremental treadmill test performed wearing elastomeric garment or placebo.

	Elastomeric garment						Placebo garment					
	*Stage 8* * * *km/h*	*Stage 10* * * *km/h*	*Stage 12* * * *km/h*	*Stage 14* * * *km/h*	*Stage 16* * * *km/h*	*Stage 18* * * *km/h*	*Stage 8* * * *km/h*	*Stage 10* * * *km/h*	*Stage 12* * * *km/h*	*Stage 14* * * *km/h*	*Stage 16* * * *km/h*	*Stage 18* * * *km/h*
*Global RPE*	1.85 (0.69)^a^ ^,^ ^b^ ^,^ ^c^ ^,^ ^d^ ^,^ ^e^	3.31 (0.97)^b^ ^,^ ^c^ ^,^ ^d^ ^,^ ^e^	4.38 (1.02)^c^ ^,^ ^d^ ^,^ ^e^	6.08 (0.93)^d^ ^,^ ^e^	7.38 (1.05)^e^	8.69 (1.03)	1.46 (0.66)^a^ ^,^ ^b^ ^,^ ^c^ ^,^ ^d^ ^,^ ^e^	3.23 (0.96)^b^ ^,^ ^c^ ^,^ ^d^ ^,^ ^e^	4.69 (0.85)^c^ ^,^ ^d^ ^,^ ^e^	6.15 (0.92)^d^ ^,^ ^e^	7.62 (1.03)^e^	8.54 (1.07)
*Respiratory RPE*	2.00 (0.71)^a^ ^,^ ^b^ ^,^ ^c^ ^,^ ^d^ ^,^ ^e^	3.38 (0.96)^b^ ^,^ ^c^ ^,^ ^d^ ^,^ ^e^	4.92 (0.95)^c^ ^,^ ^d^ ^,^ ^e^	6.54 (0.97)^d^ ^,^ ^e^	7.69 (0.95)^e^	8.85 (0.98)	1.69 (0.63)^a^ ^,^ ^b^ ^,^ ^c^ ^,^ ^d^ ^,^ ^e^	2.92 (0.76)^b^ ^,^ ^c^ ^,^ ^d^ ^,^ ^e^	4.31 (0.85)^c^ ^,^ ^d^ ^,^ ^e^	5.77 (0.93)^d^ ^,^ ^e^	7.54 (0.97)^e^	8.92 (0.99)

Results are presented as mean ± standard deviation.

^a^ Significant differences (*p* < 0.05) compared with Stage 10 km/h.

^b^ Significant differences compared with Stage 12 km/h.

^c^ Significant differences compared with Stage 14 km/h.

^d^ Significant differences compared with Stage 16 km/h.

^e^ Significant differences compared with Stage 18 km/h; RPE: rating of perceived exertion.

## 4 Discussion

This is the first study to compare the effects of a new sports garment that incorporates elastomeric technology and a placebo garment on physiological and psychological responses in healthy trained adults during an incremental treadmill test. The main finding was that performing the incremental treadmill test with the elastomeric garment did not interfere with the performance (time to exhaustion) and significantly improved neuromuscular activation of the pectoralis major and triceps, and the capillary blood lactate clearance compared to the placebo garment. Considering that no previous study analyzed the effect of running wearing an elastomeric garment that covers the torso and arms, it was necessary to discuss our findings with other studies that analyzed the effect of any other type of external load during human locomotion (e.g., walking poles, compressive garments, hand weights).

First, the use of the elastomeric garment did not interfere with the performance (time to exhaustion) during an incremental treadmill test. Since there were no significant differences in the time to exhaustion, it is suggested that the elastomeric garment does not compromise physical performance during progressive running. This is of great importance since the objective of adding a garment is to improve performance and optimize positive physiological responses during training.

Regarding muscle activation, the elastomeric garment allowed participants to achieve greater muscle activation in the long head of the triceps during the first two stages (10 km/h, *p* = 0.01, *d* = 0.41; 12 km/h, *p* = 0.04, *d* = 0.28) and in the clavicular bundles of the pectoralis major during the last two stages (16 km/h, *p* = 0.01, *d* = 0.47; 18 km/h, *p* = 0.02, *d* = 0.55) compared to the placebo garment. These results for the triceps brachii are consistent with numerous studies (Knight and Caldwell, 2000; Sugiyama et al., 2013; Pellegrini et al., 2015) that examined differences in muscle activation and physiological responses between conventional treadmill and Nordic walking with an external upper body load (e.g., walking poles). These authors reported 16-fold (Pellegrini et al., 2015) and 3-fold increases (Knight and Caldwell, 2000; Sugiyama et al., 2013) in triceps brachii muscle activation during Nordic walking compared to standard walking. On the other hand, Hamner et al. (Hamner et al., 2010) examined the kinematics of running (constant speed of 3.96 m/s [≈14 km/h]) in a single healthy subject by analyzing 41 anatomical points (including the triceps brachii). They stated that the arms did not substantially contribute to either propulsion or support during running, with a maximum contribution of less than 1% of the maximum horizontal and vertical center-of-mass accelerations (Hamner et al., 2010). While the study by Hamner et al. (Hamner et al., 2010) provided a first approximation to the muscular contribution of the upper extremities in the propulsion and support phases during running, the results may not represent a general running strategy because they only analyzed one participant at a constant speed of 3.96 m/s (≈14 km/h). Finally, a possible reason for the increased activation of the long head of the triceps could be the additional 8 kg provided by the elastomeric garment. Considering the elastic capacity of the garment, it may be possible that the assistance capacity of the elastomeric garment at moderate speeds (8–10 km/h) is lower, causing greater work of the shoulder extensors, and therefore, greater muscle activation (e.g., the long head of the triceps brachii as it originates from the infraglenoid tubercle of the scapula) (Landin and Thompson, 2011). However, there were no significant differences in muscle activation in the later stages of the test, possibly because of the inertia of the movement that may be generated by a greater assistance capacity of the elastomeric garment at higher speeds (14 km/h, 16 km/h, and 18 km/h). This would be caused by a greater accumulation of elastic energy with increased inertia of trunk rotation. This study discovered greater muscle activation of the pectoralis major when using the elastomeric garment at the fastest test speeds (16 and 18 km/h), as indicated by statistical significance (*p* = 0.01, *d* = 0.47; *p* = 0.02, *d* = 0.55, respectively). Only a few studies reported data on pectoralis major activation during running (Milligan et al., 2014; Macadam et al., 2018). Thus, we propose that the increased resistance added by the elastomeric garment to the shoulder flexion during the higher stroke cadence at the highest speeds, compared to the lowest, may stimulate greater muscle activation of the pectoralis major.

Although there were significant increases in the activation of the rectus abdominis and anterior deltoid caused by the increase in speed during the test, it should be noted that there were no significant differences compared to the placebo garment. Regardless of wearing the elastomeric garment or the placebo, the evolution of muscle activation during the test coincided with previous studies (White and McNair, 2002). These authors indicate that the increase of trunk muscle activity during walking and slow running is low (0%–40%). In our study, from 10 to 12 km/h, it increased by 20.85% wearing the elastomeric garment, and by 7.56% wearing the placebo garment. According to Saunders et al. (Saunders et al., 2004), abdominal EMG increases with increasing running speed. In our study, from 10 to 18 km/h, activation of the rectus abdominis increased by 40.08% wearing the elastomeric garment, and by 39.61% wearing the placebo garment. Literature indicates that running involves a series of unilateral hip flexion and extension movements that can exert considerable destabilizing torque on the trunk (Saunders et al., 2004; Saunders et al., 2005; Behm et al., 2009). Therefore, one reason that could explain the greater activation of the rectus abdominis could be its stabilizing role against the lower extremity reaction moments and forces (Pellegrini et al., 2015). To counteract these forces, the abdominal musculature through the eccentric contraction phase decelerates lumbopelvic movement by increasing muscle activation levels (Saunders et al., 2005). Another aspect to consider is the increased number of exhalations during running (Saunders et al., 2004). Non-significant differences existed between the elastomeric garment and the placebo in the rectus abdominis activation. A possible reason may be that, according to the location of the elastomers, the elastomeric garment does not resist the direction of core movements (Kavcic et al., 2004) and, therefore, the effect of both garments on core muscle activity was the same. Regarding muscle activation of the deltoid during walking and running, the literature distinguishes two distinct approaches. Previous research suggests that the deltoid, together with the pectoralis major, is responsible for moving and stabilizing the upper arm at the glenohumeral joint to flex the shoulder during walking and running (Smoliga et al., 2010). However, other studies propose that the arm swing is mostly a passive response to the forces exerted on the torso by the leg swing and that the deltoid only acts to stabilize the shoulder through co-contraction or eccentric contraction, limiting its role in driving arm swing (Pontzer et al., 2009).

When capillary blood lactate is analyzed at the fingertip or earlobe, lactate concentration is an integrated indicator of (i) the release of lactate from muscle to blood, (ii) the distribution of blood lactate between the systemic circulation and blood tissue, and (iii) the uptake or depletion of lactate by muscles and other organs, such as the liver or heart (Sperlich et al., 2014). The results of our study indicate that the use of an elastomeric garment, which can also act as a compression garment, aids the recovery process by reducing the capillary blood lactate concentration after exercise. The use of the elastomeric garment (which can also act as a compression garment) generated significantly lower blood lactate immediately after the test (*p* = 0.001; *d* = 0.68) and after 5 min of rest (*p* < 0.001; *d* = 1.00) compared to the placebo. These differences are consistent with previous studies that compared the effect of a compressive garment *versus* a placebo garment during the execution of different physical exercise tests (Lovell et al., 2011; Sperlich et al., 2014). Other studies reported no influence of compression garments on lactate levels (Hamner et al., 2010; Sugiyama et al., 2013). Potential explanations for the lactate variations are that, during recovery, skeletal muscles facilitate blood return by rhythmic muscle contractions that create oscillations in intramuscular pressure, which increases blood flow and venous return (Notarius and Magder, 1996). Likewise, increased blood flow is thought to enhance lactate removal from exercising muscles, which facilitates faster redistribution to alternative sites such as the heart, liver, and non-working muscles (Menzies et al., 2010). Another possible explanation may be linked to arteriolar-venular communication. Hester and Hammer (Hester and Hammer, 2002) suggested that compression may increase shear stress and decrease the venular lumen, which would lead to the release of endothelial dilators and eventually generate dilation in the neighboring blood vessels (Reneman et al., 2006). The study by Rider et al. (Rider et al., 2014), suggested that the lower blood lactate concentration observed after wearing a compressive garment was the result of a shorter time under fatigue. However, considering that in our study, there were no significant differences between the time to exhaustion in both conditions (i.e., elastomeric garment and placebo garment), this is not a plausible explanation for our results. We did not find differences in the intra-test capillary blood lactate concentration between wearing the elastomeric garment and wearing the placebo garment. This is in contrast with the study by Lovell et al. (Lovell et al., 2011), who reported lower blood lactate during a running test performed wearing a compressive garment for the lower limbs compared to common running shorts. The lack of difference in the intra-test measurement in our study could be that the accumulated load (approx. 4 mins at 8–10 km/h) is insufficient to elicit significant acute metabolic changes in active, healthy, young participants with previous training experience. Another explanation for the non-significant differences in the intra-test lactate could be that the elastomeric garment is compressing the trunk and not the active muscles (lower limbs). In short, elastomeric garments that can also act as compression garments seem to maintain similar lactate levels during running, but significantly reduce lactate concentration immediately after and 5 minutes post-exercise.

The heart rate increased during the incremental treadmill test both with the elastomeric and placebo garments (Table 2). However, there were no differences in heart rate between the garments. This agrees with other studies in which similar conditions were applied (Ali et al., 2010; Gene-Morales et al., 2023). These findings could be related to the area of the body covered by the compression garment, as other studies have shown significant increases in heart rate when a full-body compression garment was worn (Sear et al., 2010), whereas compression garments that partially covered the body (e.g., torso, arms, thighs, calves) did not demonstrate modifications in heart rate (Ali et al., 2010; Mizuno et al., 2017), as was in our study. We did not find significant differences in systolic and diastolic blood pressure between conditions (Table 2), which is in accordance with other studies that did not report modifications in blood pressure after the application of compressive garments (Venckūnas et al., 2014; Book et al., 2016). Blood pressure is regulated by a central homeostatic mechanism, including cardiac output, blood volume, and peripheral resistance (Huonker et al., 1996). Previous studies have suggested that wearing graduated compression garments during and after exercise facilitates greater venous return, thus improving cardiac output and stroke volume, which in turn could improve blood flow (Liu et al., 2008). The studies by de Glanville et al. (de Glanville and Hamlin, 2012) and Liu et al. (Liu et al., 2008) used a pressure of approximately 10–14 mmHg at the ankle (Liu et al., 2008), calf, and thigh (de Glanville and Hamlin, 2012) without reporting significant differences between the conditions. Although previous studies have been successful in measuring specific pressures that generate changes in blood pressure (de Glanville and Hamlin, 2012), many other investigations have not analyzed the effect of applying a specific pressure to a specific region of the body. This association between mechanical pressure and blood pressure could explain certain discrepancies in the results, since in many investigations, mechanical pressure values were either too low to elicit the desired effects (Liu et al., 2008; de Glanville and Hamlin, 2012) or were not measured, as was the case in our study. Ultimately, the findings of this study indicate that the occlusion capacity of the elastomeric garment does not compromise the cardiovascular health of the participants, demonstrating its safety, in contrast to other compressive garments that are hemodynamically more demanding (Sear et al., 2010; Leoz-Abaurrea et al., 2016).

The incremental treadmill running protocol employed in this study did not result in significant differences between the conditions in global and respiratory effort perception. These results are in line with those of Ali et al. (Ali et al., 2010) and Sperlich et al. (Sperlich et al., 2010; Sperlich et al., 2014), who analyzed the effect of compressive garments on the lower and upper extremities in long-distance and sprint running tests. Another study by Vincent et al. (Vincent et al., 2018) stated that adding an external resistance through hand-held water bottles during running could elicit a higher perception of muscle exertion. Oppositely, we found that adding an external resistance through the elastomeric garment did not modify effort perception. Therefore, we consider these results to be positive since the elastomeric garment generated a series of improvements in physiological parameters without the participants perceiving the effort as more exhausting.

In conclusion, the elastomeric garment worn during the incremental treadmill test allowed participants to achieve greater muscle activation in determinate muscles and speeds without perceiving the exercise as more strenuous. Additionally, lower post-exercise capillary blood lactate levels were obtained with the elastomeric garment. Finally, it was demonstrated that using the elastomeric garment to run is as safe as running with a placebo garment regarding cardiovascular parameters. All these milestones mostly indicate that the elastomeric garment does not impair the running performance and could optimize determinate physiological parameters.

### 4.1 Limitations and future research

It is critical to acknowledge that the outcomes of this study are constrained by the specific dependent and independent variables analyzed. Consequently, further research that includes participants with diverse experience, physical fitness levels, and from both sexes is required. New variables could be examined: (i) maximal oxygen consumption, to estimate the actual metabolic stress depending on the fitness status of each participant; (ii) creatine kinase values and changes in muscle volume, stiffness, and circumference to assess muscle damage; (iii) the effects of the elastomeric garment on the skin to analyze thermoregulation; and (iv) aspects linked to sports biomechanics, such as running technique, joint angles, oscillatory patterns between limbs and muscles, and range of motion. Similarly, it would be interesting to analyze performance and muscle function in tests related to agility and jumping.

## Data Availability

The raw data supporting the conclusion of this article will be made available by the authors, without undue reservation.
